# Seed storage behaviour of tropical members of the aquatic basal angiosperm genus *Nymphaea* L. (Nymphaeaceae)

**DOI:** 10.1093/conphys/coz021

**Published:** 2019-05-10

**Authors:** Emma L Dalziell, Bryn Funnekotter, Ricardo L Mancera, David J Merritt

**Affiliations:** 1 Kings Park Science, Department of Biodiversity, Conservation and Attractions, Kings Park, WA, Australia; 2 School of Biological Sciences, The University of Western Australia, Crawley, WA, Australia; 3 School of Molecular and Life Sciences, Curtin University, Bentley, WA, Australia; 4 School of Pharmacy and Biomedical Sciences, Curtin Health Innovation Research Institute, Curtin University, Perth WA, Australia

**Keywords:** Basal angiosperms, comparative longevity, cryogenic storage, desiccation tolerance, moisture content, storage temperature

## Abstract

Eighteen native species of *Nymphaea* (waterlilies) inhabit a range of freshwater wetlands in northern Australia, which are threatened by increased development and the potential impacts of climate change. To investigate conservation seed banking of these vulnerable species, we aimed to characterize their seed storage physiology by determining (i) seed desiccation tolerance and (ii) the effects of moisture content and storage temperature on seed germination and viability. Seeds of *N. immutabilis*, *N. lukei*, *N. macrosperma* and *N. violacea* (including multiple collections of three species) were placed in experimental storage at a range of temperatures (25°C, 5°C, −20°C and −190°C) following pre-equilibration at different RHs (15%, 30%, 50%, 70% or 95%). Seeds were also experimentally aged at 60% RH and 45°C to assess comparative longevity. We found seeds of all species to be desiccation tolerant. However, the responses of seeds to experimental storage conditions were complex and variable between species and collections of the same species, and seeds of many species/collections were short-lived across many of the storage treatments. In many cases decreasing storage temperature did not increase longevity. Additional protocol development is necessary before we can have confidence that *ex situ* seed banking is a viable long-term germplasm conservation strategy for *Nymphaea*.

## Teaser

The *ex situ* seed storage behaviour of four species of Australian waterlilies (*Nymphaea*) was investigated. Seeds of all species were found to be desiccation tolerant; however, most collections lost viability rapidly, even under standard genebank conditions (i.e. 15% RH and 15°C).

## Introduction

The Nymphaeaceae (Nymphaeales) comprises *c.* 80 species of aquatic, basal angiosperms, of which the cosmopolitan genus *Nymphaea* is the most speciose and widely distributed (Löhne *et al.*, 2008). In Australia, 18 species of *Nymphaea* occupy a range of ephemeral and perennial wetlands in the monsoonal north (Jacobs and Hellquist, 2006, 2011). Many of these wetland habitats are threatened by increasing development, weeds, feral animals and impacts of climate change through altered hydrologic regimes and saltwater intrusion in low-lying coastal floodplains (BMT WBM, 2010; Pettit *et al.*, 2016). Whilst there has been a concerted effort to preserve a number of freshwater wetlands within reserves, threatening processes are complex and the implementation of *in situ* management strategies alone is unlikely to mitigate all risks. Therefore, the investment in other conservation measures, such as *ex situ* seed banking, forms an integral component of biodiversity management for the continued preservation of important and at-risk plant species in this region, including *Nymphaea*.

Seed banking is a widely applicable method of conserving plant germplasm (Hay *et al.*, 2000). However, the ability to dry seeds to low moisture content and store at low temperatures underpins successful long-term seed banking. Seeds are commonly classified into one of three categories of storage behaviour based on their degree of desiccation tolerance and the survival of storage at sub-zero temperatures (Hong and Ellis 1996). However, accumulating data for wild species point to at least five categories of storage behaviour, reflecting a continuum of desiccation tolerance/sensitivity and longevity amongst species (Walters 2015). In contrast with terrestrial species, the storage behaviour and longevity of seeds from aquatic plants are less well known (Hay *et al.*, 2000). Desiccation sensitivity is often found in seeds shed into wet environments (Wyse and Dickie 2017), and early hypotheses proposed basal angiosperms that evolved in mesic habitats could prove to be less resilient to desiccation and subsequent storage than species of (seasonally) dry environments (Dickie and Pritchard 2002; Tweddle *et al.*, 2003). However, in a study of seeds from 87 aquatic species, predominantly from temperate aquatic environments, ~75% of species were considered to have orthodox seeds (Hay *et al.*, 2000), and seeds of the basal, aquatic *Trithuria* genus (*T. austinensis*, *T. bibracteata* and *T. submersa*) are also considered desiccation-tolerant and orthodox (Tuckett *et al.*, 2010b). Within the *Nymphaea*, evidence of seed desiccation tolerance (or sensitivity) is sparse, and conflicting. Seeds of the temperate northern hemisphere species *N. alba* and *N. odorata* are considered desiccation sensitive (Guppy, 1897; Else and Riemer 1984; Smits *et al.*, 1989; Hay *et al.*, 2000; Estrelles *et al.*, 2004), whereas seeds of *N. gigantea*, a tropical species, are thought to be desiccation tolerant (Ewart, 1908). Of the 12 records for *Nymphaea* retrieved from the Kew Seed Information Database (Royal Botanic Gardens Kew, 2018), only five species have been assigned to a storage behaviour category; seeds of two species are considered orthodox (*N. gigantea* and *N. maculata*), one species is recalcitrant (*N. alba*) and two species are listed as ‘uncertain’ (*N. odorata* and *N. tuberosa*).

If seeds of tropical Australian *Nymphaea* prove to be desiccation tolerant, then seed longevity becomes an important consideration for the ongoing management of *ex situ* collections. Seed longevity can vary between species by up to four orders of magnitude under the same storage conditions (Probert *et al.*, 2009; Mondoni *et al.*, 2011; Merritt *et al.*, 2014; Satyanti *et al.*, 2018), and the effective curation of seed collections requires knowledge of the variation in longevity between species, as well as the identification of potentially short-lived seeds in particular. Correlates between seed longevity and their environment of origin have been demonstrated, with seeds originating in hot, dry climates often longer-lived than those originating from moist, temperate regions (Walters *et al.*, 2005; Probert *et al.*, 2009). Seeds of aquatic species of temporary pools in south-west Australia, including *Trithuria*, have broadly similar longevity under controlled aging conditions and are shorter-lived when compared with terrestrial Australian species (Probert *et al.*, 2009; Tuckett *et al.*, 2010a, 2010b). Significant advancements have been made in understanding the storage requirements of wild species in the past two decades (Hay and Probert 2013; Walters, 2015). However, the successful storage of seeds from wild species is still challenging, due predominantly to the sheer scale of genetic and geographic diversity wild species represent, and the inherent variation this imparts (Walters, 2015).

We aimed to (i) determine whether seeds from the genus *Nymphaea* from northern Australia were tolerant of desiccation, equivalent to standard genebank procedures (i.e. drying to 15% RH, equivalent to an internal seed moisture content of 3–7%), (ii) examine the effects of storage temperature and seed moisture content on the viability of seeds over a 12-month storage period and assess whether conventional seed storage techniques would be appropriate for the *ex situ* conservation of tropical *Nymphaea* and (iii) evaluate the comparative longevity of seeds under accelerated aging conditions. We also examined low temperature storage behaviour via differential scanning calorimetry (DSC). As it is recognized that seed longevity can vary substantially within species (Walters *et al.*, 2005, Kochanek *et al.*, 2009; Probert *et al.*, 2009), we assessed where possible multiple, geographically and/or temporally diverse collections of the following three species: *N. lukei*, *N. macrosperma* and *N. violacea*.

## Materials and methods

### Species selection, seed collection and seed quality

**Figure 1:**
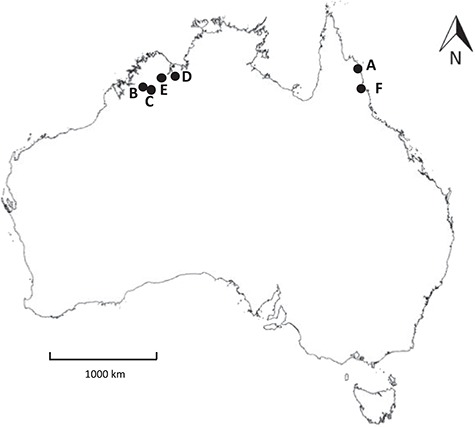
Collection locations of *Nymphaea* species used in this study: (A) *N. immutabilis* NI1; (B) *N. lukei* NL1; (C) *N. lukei* NL2; (D) *N. macrosperma* NM1 and NM2, *N. violacea* NV1; (E) *N. violacea* NV2 and NV3 (two separate collections made from different populations, located ~25 km apart); (F) *N. violacea* NV4.

Seeds of *N. immutabilis* S.W.L. Jacobs (collection number NI1), *N. lukei* S.W.L. Jacobs & Hellq (two separate collections, NL1 and NL2), *N. macrosperma* Merr. & L.M. Perry (NM1 and NM2) and *N. violacea* Lehm. (NV1, NV2, NV3, NV4) were collected from northern Australia between May 2012 (NL1, NM1 and NV1) and August 2013 (all other collections; Fig. 1). Seed collections were made either from geographically distinct populations (i.e. at least 25 km apart), or in the case of NM1 and NM2, were collected from the same geographic location over the two separate years. For *N. lukei*, *N. macrosperma* and *N. violacea* (NV1, NV2 and NV3) whole, mature fruits were collected in the field and placed in sealed bags with ~30 mL of water and transported to Kings Park, Perth, Western Australia. Fruits were left inside the bags at room temperature (*c.* 24°C) to allow seeds to naturally dehisce. Seeds were then rinsed through brass sieves (710 μm–2.36 mm, Endecotts, London, UK) to separate any remaining plant material. For seeds of *N. immutabilis* and one collection of *N. violacea* (NV4), whole mature fruits were collected, and the seeds cleaned manually from the fruit in the field. Cleaned seeds were then placed in sealed bags containing a sheet of moistened paper towel prior to transport.

To ensure all seeds were filled prior to experimentation, seeds from all collections were patted dry with paper towel and X-rayed (Faxitron Specimen Radiography System MX-20 Cabinet, Tucson, Arizona). Empty seeds were identified and removed, until the seed fill was > 99%. Seeds were kept damp prior to the commencement of experiments, which occurred within 4 weeks of collection, with the exception of seeds used for moisture sorption isotherms and DSC.

### Seed germination and post-germination viability assessment

Seed germination tests were used in a number of the experiments presented below, and all germination tests were conducted in the same manner, unless stated otherwise. Seeds were surface sterilized in 2% (w/v) calcium hypochlorite solution with two drops of surfactant (Tween 80®, Hurst Scientific, Perth) under partial vacuum (10 minutes on/off/on at −80 kPa) for 30 minutes, and then were rinsed three times in sterile water. Four replicates of 100 seeds were placed in 10 ml plastic tubes (TechnoPlas, Australia) with 10 ml of sterile water and incubated for 8 weeks at 35°C under a daily 12 h/12 h photoperiod of 30 μmol m^−2^ s^−1^, 400–700 nm cool white fluorescent light (Dalziell *et al.*, 2018). Seeds were scored every 2–3 days for germination, which was defined as emergence of the hypocotyl ≥2 mm. To assess viability after the 8-week germination period, all ungerminated seeds were subjected to a cut-test, where seeds were cut in half lengthways and firm, white and turgid embryos were considered viable (ISTA, 2017). Where seeds could not be immediately considered viable (e.g. soft or discoloured tissue) or appeared to be non-viable, seeds were assessed with tetrazolium chloride (TZ), whereby seeds were placed cut-side down on glass filter paper irrigated with 1% TZ buffered to pH 7 with a phosphate buffer (KH_2_PO_4_ and Na_2_HPO_4_) for 24–36 hours until viable embryos stained a uniform red.

### Seed desiccation tolerance

The moisture status of freshly collected seeds was determined using a portable hygrometer (Rotronic HygroPalm, HP AW1, Switzerland), by measuring the equilibrium relative humidity of a subsample of seeds extracted from fruits within a few minutes of collection. Germination testing was then undertaken on a sample of freshly collected seeds, prior to drying. To assess seed desiccation tolerance, freshly collected seeds (2013 collections only) were dried for 28 days in a controlled environment facility at 15% RH at 15°C and germinated (as above). To assess the moisture content of dried seeds, three replicates of either 0.05 g of seeds (*N. lukei* and *N. violacea*), or 25 seeds (*N. immutabilis* and *N. macrosperma*), were weighed then dried in an oven at 103°C for 24 hours (ISTA, 2017), and moisture content calculated gravimetrically, on a fresh weight basis.

### Effects of storage temperature and moisture content on seed germination and viability

To test the effect of temperature and moisture conditions during storage on seed germination and viability, a full factorial experimental design was implemented for the 2013 seed collections, and consisted of five experimental RH treatments, 15%, 30%, 50%, 70% and 95% RH; four storage temperatures, 25°C, 5°C, −20°C and ≤−190°C (vapour phase liquid nitrogen; LN); and three time periods in storage, 0, 6 and 12 months. Due to the limited number of seeds from the 2012 collections, seeds of *N. violacea* NV1, *N. macrosperma* NM1 and *N. lukei* NL1 were only stored at −20°C and ≤−190°C. To adjust moisture content prior to storage, freshly collected seeds of all species and collections were sealed inside porous nylon mesh bags and stored inside polycarbonate electrical enclosure boxes (NHP, Fibox, Australia) containing non-saturated solutions of lithium chloride (LiCl) at the five RHs at 20°C for 2 weeks (Hay *et al.*, 2008). To ensure seeds had equilibrated to the experimental RHs, a subsample of seeds from each collection were removed from the RH boxes, tested with the hygrometer and then discarded. For ease of interpretation, seed moisture content is hereafter expressed based on pre-storage equilibrium RH. Seeds stored at 25°C, 5°C and −20°C were hermetically sealed inside two laminated aluminium foil bags prior to storage at the appropriate temperature, whilst seeds stored in LN were sealed inside 1 ml Nunc CryoTube™ vials (Thermo Fisher Scientific, USA) and then plunged directly into LN vapour. Four replicates of 50 seeds of *N. lukei* and *N. violacea* and one replicate of 50 seeds of *N. macrosperma* and *N. immutabilis* were extracted from each storage temperature at 1, 6 and 12-month intervals and tested for germination. Fresh seed germination, prior to drying (as assessed when testing for desiccation tolerance), acted as the pre-storage control.

### Comparative longevity

The comparative longevity of the 2013 collections of *Nymphaea* seeds was determined using the experimental protocols described by Probert et al. (2009). Seeds were placed inside sealed mesh bags and enclosed inside an air-tight electrical enclosure box (NHP, Fibox, Australia) and hydrated above a non-saturated solution of LiCl (370 g L^−1^) creating an RH of 47% at 20°C for 14 days (Hay *et al.*, 2008). Seeds were then transferred to a second electrical enclosure box above a non-saturated solution of LiCl (300 g L^−1^) creating an RH of 60% and stored in an oven at 45°C. Four replicates of 50 seeds were removed from the experimental enclosure box at 0, 1, 2, 5, 9, 20, 37, 56, 75, 100 and 125 days and tested for germination; however viability (via TZ staining) was not assessed. A T-tec data logger (Temperature Technology, Adelaide) was used to monitor the RH and temperature inside the electrical enclosure box to ensure a constant RH and temperature was maintained during the experiment.

### Moisture sorption isotherms

Moisture sorption isotherms were determined using seeds of the 2013 collections stored at 15% RH and 15°C for at least 4 months. Seeds were placed at 5%, 15%, 30%, 50%, 70%, 85%, 90% and 95% RH at 20°C for 2 weeks. The 15–95% RH environments were created using solutions of LiCl (Hay *et al.*, 2008), whilst the 5% RH environment was created using a saturated solution of ZnCl_2_ (Vertucci and Roos 1993). To ensure seeds had equilibrated to the correct RH after 2 weeks, a subsample of seeds of each species was tested with a hygrometer. Seeds (three replicates of ≥0.05 g for *N. lukei* and *N. violacea*, or three replicates of 25 individual seeds for *N. immutabilis* and *N. macrosperma*) were then weighed and moisture content was calculated gravimetrically after drying at 103°C for 24 hours (ISTA, 2017) on a dry weight basis.

### Seed lipid content

Seed lipid content for the 2013 collections was determined using the methods of Merritt et al. (2003). Three replicates of ~ 1 g seed (at air-dry moisture content) were homogenized in methanol:chloroform (1:2 by volume) using a mortar and pestle. The homogenate was then transferred to clean 10 mL tubes and centrifuged at 12 000 rpm for 15 minutes. The supernatant was washed with 5 mL of 0.7% (w/v) NaCl to remove all non-lipid material and centrifuged again at 10 000 rpm for 10 minutes. The organic layer was separated and dried under a constant flow of N_2_ for 1 hour, and the residual lipids were assessed gravimetrically and expressed as a percentage of the seed fresh weight.

### DSC

DSC (Perkin-Elmer DSC 8000, calibrated with indium, equipped with a Perkin-Elmer CLN_2_ controlled liquid nitrogen cooling system) was used to determine the unfrozen moisture content of whole seeds of all species. Prior to analysis, seeds were equilibrated at 20°C for 14 days at 15%, 30%, 50%, 70%, 85%, 90%, 95% and 100% RH (as described above). A further sample of seeds was imbibed on irrigated glass filter paper for 48 hours prior to analysis. Empty, hermetically sealed (Perkin-Elmer, USA crucible sealing press) aluminium crucibles (Perkin-Elmer, USA) were used to obtain the baseline curvature on the DSC instrument. Three replicates of seeds from each species and RH combination were individually sealed within an aluminium crucible and cooled to −50°C at a rate of 10°C min^−1^ and rewarmed to 30°C at a rate of 50°C min^−1^. Following DSC analysis, crucibles were punctured and dry weights calculated after drying at 103°C for 24 hours (ISTA, 2017). Thermal transitions were measured as endothermic (melting) peaks in the baseline and enthalpy of the transitions measured in J g^−1^ d.wt. from the area above the baseline using Pyris software (Perkin-Elmer, USA version 10.1.0.0412).

**Figure 2:**
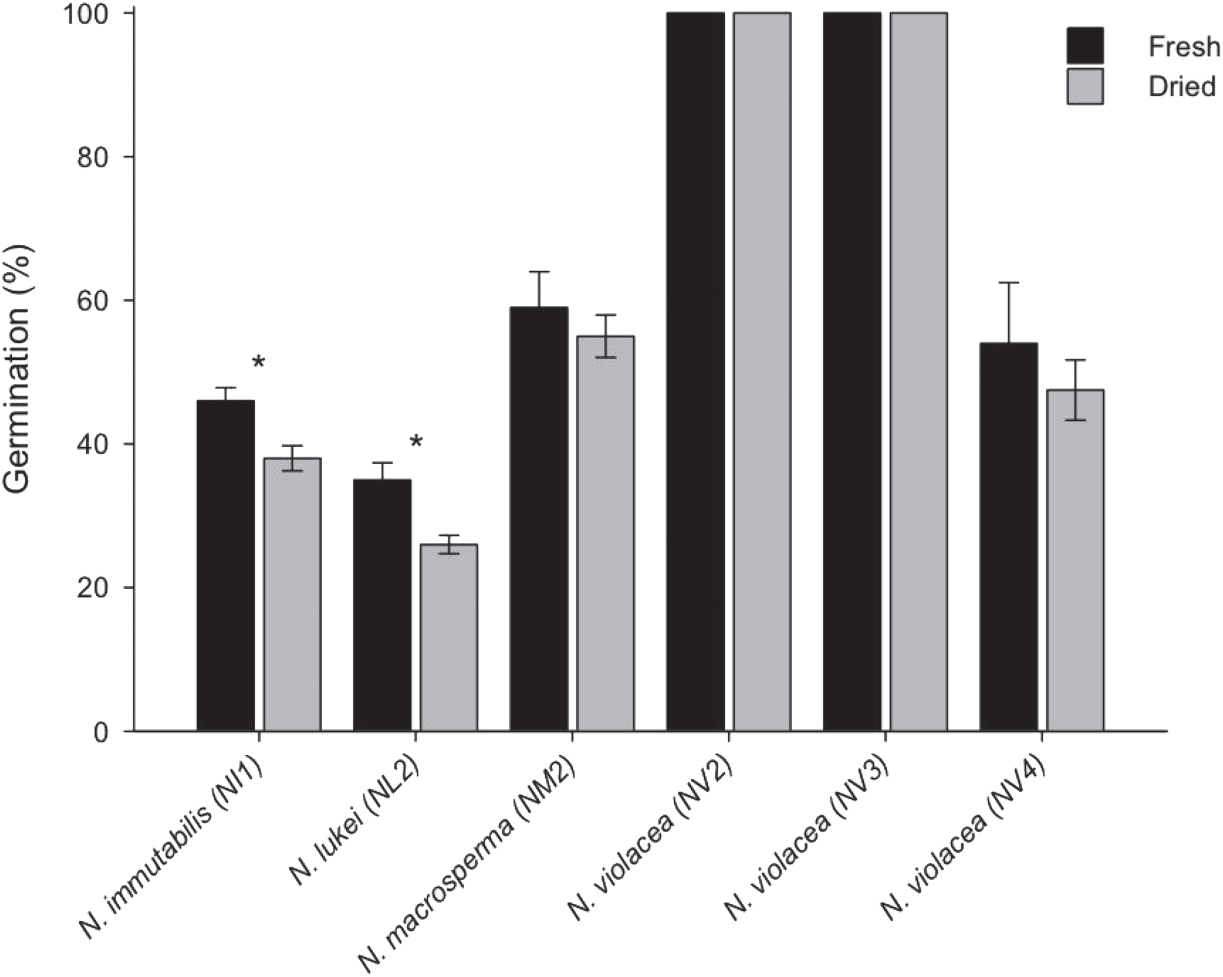
Germination of fresh and dried (15% RH and 15°C) seeds of *N. immutabilis* (NI1), *N. lukei* (NL2), *N. macrosperma* (NM2) and three collections of *N. violacea* (NV2, NV3 and NV4). Significance codes: ^*^P < 0.05.

### Statistical analyses

Germination and viability data were analysed using logistic regression fitted with a ‘logit’ link function, with a binomial error distribution in R (R Core Team, 2017). Total germination response and viability were analysed separately across full models inclusive of all factors (seed collection, storage temperature, RH and storage time) and their interactions (two-way only). Both initial germination and viability models found significant differences across all factor terms (Supplementary Table S1). As a different number of seed collections were made for each species, further analyses were conducted within each seed collection, across all species. A Wald Chi-square test was conducted following each regression analysis to determine the significance of treatment effects for each model. Comparative longevity data were analysed in Genstat (version 12, VSN International Ltd, UK). Survival data from the comparative longevity experiments were analysed via probit analysis to estimate *P_50_* (the time for viability to fall to 50%) and to fit the seed viability equation (Ellis and Roberts 1980):

\begin{equation*} \nu=K_{i}-p/\sigma \end{equation*}

where *ν* is the viability in normal equivalent deviates (NED) of the seeds after *p* days in storage, *K_i_* is the initial viability (NED) and *σ* is time (in days) for viability to fall by 1 NED. Where germination was found to increase over the storage period (i.e. evidence of improvement in germination in the early phases of storage due to after-ripening), only data showing a decline in viability were included in the analysis. Seed survival curves were plotted in OriginPro 8 (version 8, 2010, OriginLab Corporation, MA, USA). Enthalpy data determined using the DSC were plotted against seed sample water content (% H_2_O g^−1^ d.wt.) at the various RHs. Two linear regression lines were fitted using Sigma Plot (Systat Software, San Jose, CA, USA), one based on the water contents at which no change in enthalpy was detected, the other where there was an increase in enthalpy with increasing moisture content. The unfrozen water content (UWC) was determined to be at the intersection of these two lines (Dussert *et al.*, 2001).

## Results

### Seed desiccation tolerance

Initial seed moisture content of freshly collected seeds was high, with measured RHs ranging between 90–99% RH. After 28 days of drying at 15% RH and 15°C, mean internal seed moisture content reached 5.1% in all species. For freshly collected (in 2013) seeds, germination varied substantially between species or collections, ranging from 100% for *N. violacea* NV2 and NV3, to between 50–60% for *N. macrosperma* and *N. violacea* NV4 (Fig. 2). For most species/collections, there was no significant effect of drying on germination. For seeds of *N. immutabilis* and *N. lukei*, germination of fresh seeds was 46% and 35%, respectively, and there was a small, but significant reduction in germination following drying (*P* < 0.01 and *P* < 0.04, respectively). However, cut-testing and subsequent TZ staining showed all ungerminated seeds were still viable.

**Figure 3:**
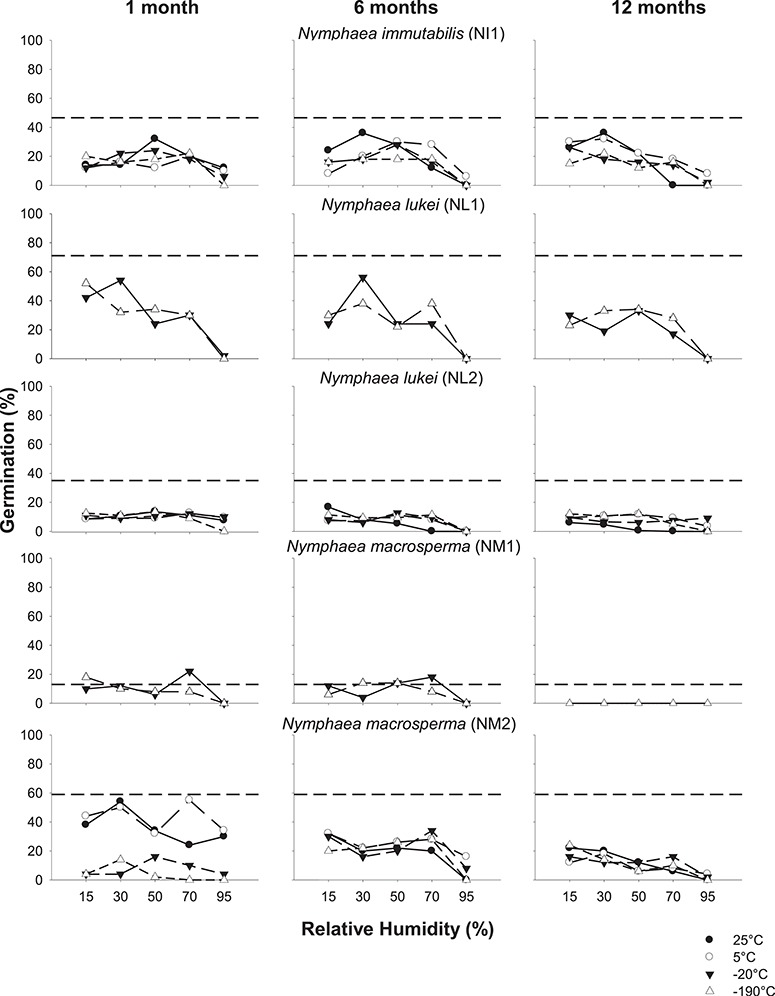
Seed germination of *N. immutabilis* (NI1), *N. lukei* (NL1 and NL2) and *N. macrosperma* (NM1 and NM2) after equilibration at 15–95% RH (at 20°C) and stored at indicated temperatures for 1, 6 or 12 months. Dashed lines indicate fresh seed germination.

**Figure 4:**
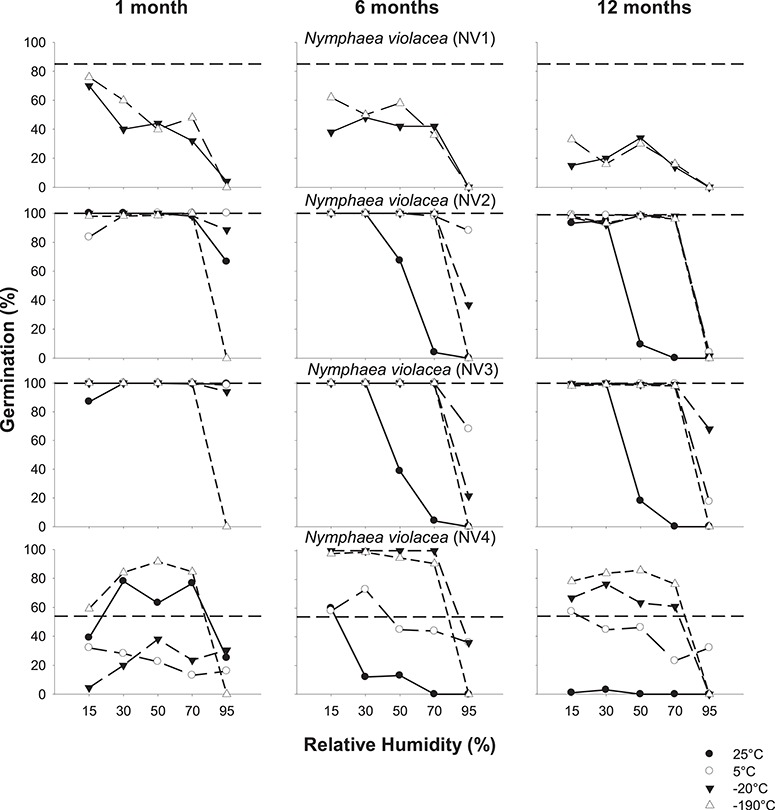
Seed germination of *N. violacea* (NV1-NV4) after equilibration at 15–95% RH (at 20°C) and stored at indicated temperatures for 1, 6 or 12 months. Dashed lines indicate fresh seed germination.

**Figure 5:**
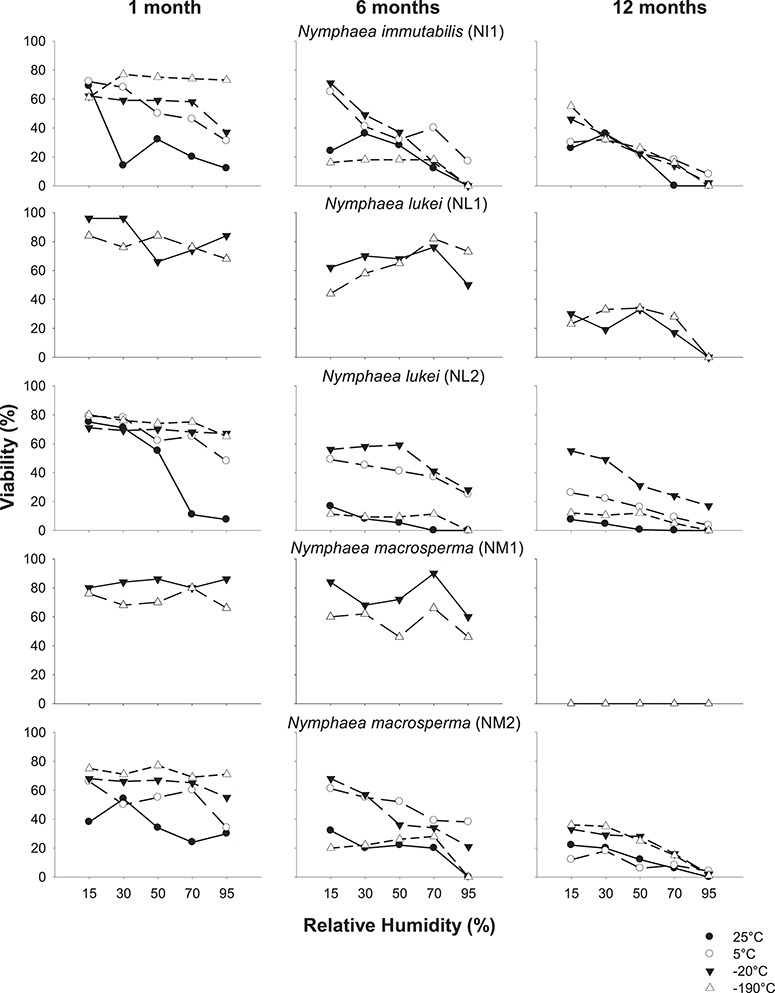
Seed viability of *N. immutabilis* (NI1), *N. lukei* (NL1 and NL2) and *N. macrosperma* (NM1 and NM2) after equilibration at 15–95% RH (at 20°C) and stored at indicated temperatures for 1, 6 or 12 months.

**Figure 6:**
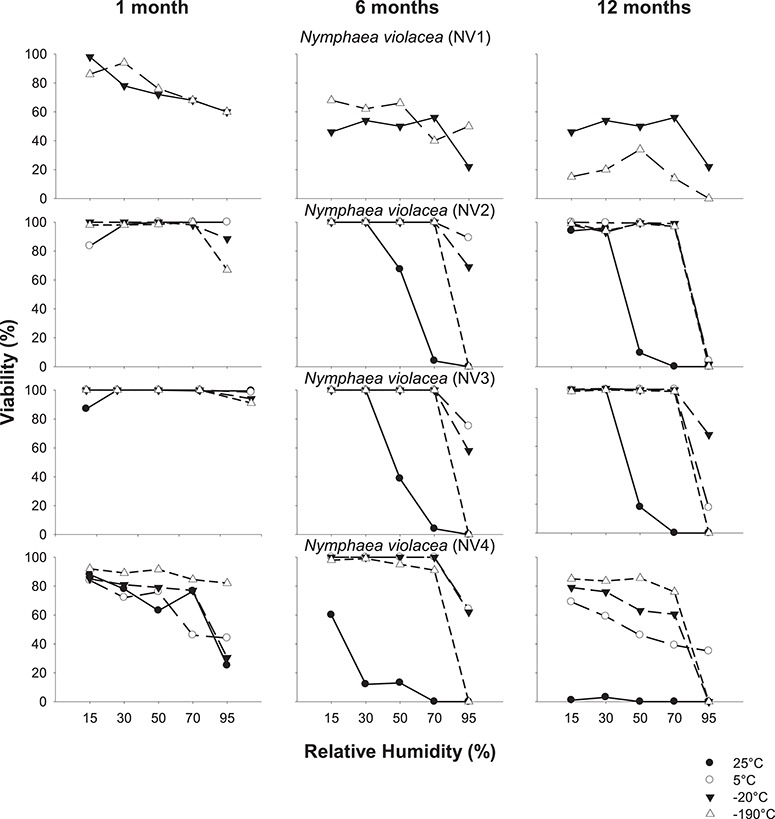
Seed viability of *N. violacea* (NV1-NV4) after equilibration at 15–95% RH (at 20°C) and stored at indicated temperatures for 1, 6 or 12 months.

### Effects of storage temperature and moisture content on seed germination

Overall, the effect of storage temperature and moisture content on seed germination differed between species, and within collections (Figs. 3 and 4; Supplementary Table S2). Across all collections, germination tended to decrease with increasing RH, temperature and time in storage (all *P* < 0.001). Within the individual seed collections, there was a marked decrease in germination in seeds of *N. immutabilis*, *N. lukei* and *N. macrosperma* under most storage conditions compared with fresh seed germination (Fig. 3). For seeds of *N. immutabilis*, germination decreased under all storage conditions, when compared with fresh seed germination (46%). This decline was most pronounced at 95% RH (*P* < 0.001; Supplementary Table S2), at all storage temperatures. The highest germination after any time in storage (34–36%) was observed for seeds stored at either 30 or 50% RH at 25°C. For fresh seeds of *N. lukei*, germination was greater in collection NL1 (71%) compared with NL2 (35%). Germination of *N. lukei* NL1 seeds decreased significantly (*P* < 0.01) with increasing RH, under both storage temperatures, whilst germination in NL2 remained low (<17%) under all RH and temperature combinations across the 12-month storage period. Fresh seed germination differed significantly between the two collections of *N. macrosperma*; NM1 germinated to 13%, whilst NM2 germinated to 59%. For NM1, seeds stored between 15–70% RH for 1–6 months showed broadly similar germination levels to fresh seed; however, no germination was detected after 12 months storage under any of the conditions. For collection NM2, germination was equivalent to fresh seeds (i.e. 50–54%) for at least 1 month for seeds at 30% RH and stored at 25°C, and for seeds at 30% or 70% RH and stored at 5°C. However, germination was significantly lower under all other storage conditions after 1 month, with seeds stored at −20°C and −190°C germinating the least. Overall, germination declined with increasing RH and time in storage.

**Figure 7:**
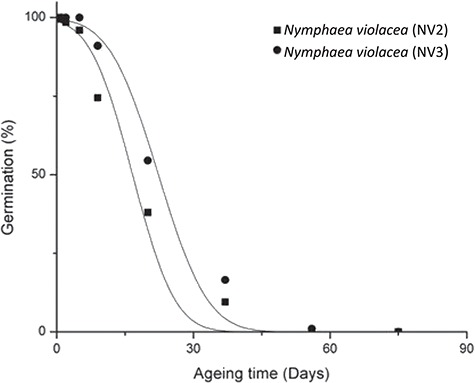
Seed survival curves of *N. violacea* (NV2, NV3) under experimental ageing conditions (60% RH and 45**°**C).

**Figure 8:**
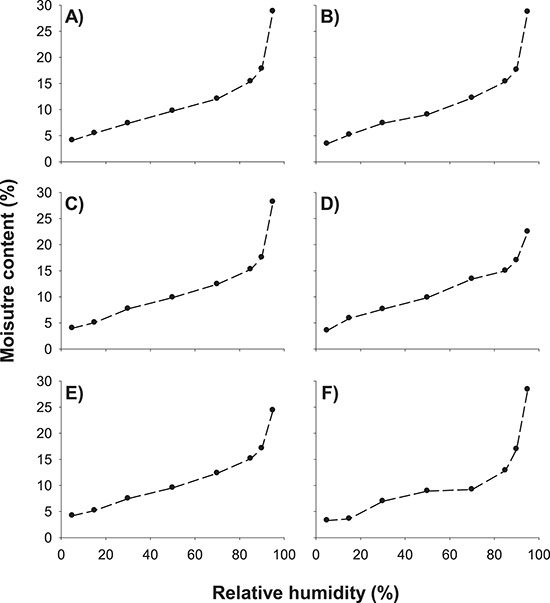
Moisture sorption isotherms for seeds of (**A**) *N. immutabilis* (NI1), (**B**) *N. lukei* (NL2), (**C**) *N. macrosperma* (NM2), (**D**) *N. violacea* (NV3), (**E**) *N. violacea* (NV3) and (**F**) *N. violacea* (NV4).

Generally, fresh seed germination of *N. violacea* seeds was high (54–100%) compared with *N. immutabilis*, *N. lukei* and *N. macrosperma*, and germination of stored seed was also generally higher over time, RH and storage temperature, compared with the other three species (Fig. 4). For seeds of NV1, germination decreased significantly with increasing RH (*P* < 0.001) and time in storage (*P* < 0.001). However, for seeds at 15% RH and stored at both −20°C and −190°C for 1 month, seed germination was still relatively high (i.e. > 70%). Collections NV2 and NV3 performed similarly in storage and, overall, maintained germinability over the 1-year storage period when at 15% RH. For seeds at 95% RH, the decrease in germination became more pronounced over time; after 1 month, only seeds stored at −190°C showed reduced germination, but after 6 and 12 months, this reduction in germination was evident at all storage temperatures. For both seed collections, seeds stored at higher moisture contents (≥50% RH) at 25°C showed the greatest decline in germination over 12 months. Germination in seeds of NV4 was inconsistent across the 12-month storage period. Whilst fresh seeds germinated to 54%, germination actually increased under some storage conditions, e.g. germination was between 59–100% for all seeds at 15–70% RH and stored at −190°C. However, germination did decrease across all temperatures when seeds were at 95% RH, and germination decreased more quickly over the 12-month storage period when stored at 25°C.

### Effects of storage temperature and moisture content on seed viability

By comparing the differences between seed germination and viability (Figs. 3 and 4 vs. 5 and 6), it is apparent for most seed collections that germination testing significantly underestimated viability, and maintenance of viability was in some cases greater than suggested by the germination data. Generally, the patterns observed in the seed germination data were relatively consistent across the viability data as, overall, germination and viability both tended to decrease at high RH and with increasing time in storage (Supplementary Tables S2 and S3). For seeds of *N. immutabilis*, viability was reduced by ≥23% after any time in storage under all conditions (all *P* < 0.001), compared with initial viability (≥99%; Fig. 5). After 6- or 12-month storage at all temperatures, viability progressively decreased with increasing RH. Viability of > 60% was maintained over the 6-month storage period in seeds at 15% RH and stored at 5°C or −20°C; however, after 12 months it had decreased to < 55%. For seeds of both collections of *N. lukei*, viability was also reduced by ≥20% after any time in storage compared to initial viability and was only maintained for 1 month at temperatures of 5°C or cooler. Viability was significantly reduced with increasing time (*P* < 0.001) and RH (*P* < 0.001). For seeds stored for 6–12 months, storage at −20°C at 15% RH resulted in the highest viability (*c*. 55%,), whilst storage at 25°C or −190°C at all RHs resulted in a loss of viability of 80–100%. Similarly, for seeds of *N. macrosperma*, viability declined over time (*P* < 0.001) and with increasing RH (*P* < 0.001), but, like seeds of *N. immutabilis* and *N. lukei*, viability was largely maintained for 1 month at −20 or −190°C regardless of RH. After 12 months, only 33–36% of seeds remained viable under storage at −20°C or −190°C and 15% RH.

For seeds from *N. violacea*, it is apparent that differences between germination (Fig. 4) and viability (Fig. 6) were less pronounced than in the other three species. Germination and viability were very similar for *N. violacea* collections NV2 and NV3, and viability was maintained over the 12-month storage period at all temperatures when at 15% or 30% RH. Viability was also maintained at 5°C, 20°C and −190°C at 50% and 70% RH, but declined over 6–12 months at 95% RH. However, at 25°C, viability declined with increasing RH above 30%. For seeds of NV1, viability was maintained between 86–98% at both −20°C and −190°C after 1 month for seeds at 15% RH. However, viability declined over time and was reduced to ≤ 56% after 12 months. For seeds of *N. violacea* NV4, the overall patterns of viability were similar to the germination patterns observed in seeds stored for 6–12 months. After 12 months, viability was > 80% for seeds stored at −190°C provided RH was ≤ 70%, but viability declined progressively with increasing RH (above 15%) at −20°C and 5°C, and no viability was evident for seeds stored at 25°C regardless of RH. For seeds of NV4 stored for 1 month, as for the other three species, viability was significantly different to germination. There was little difference between the viability of seeds at 15% RH and stored at each temperature (84–90%), but germination differed by 55% across storage temperatures. Percent viability was equal to or for the most part higher than percent germination over time, and viability showed a progressive decrease with increasing RH at all temperatures.

### Comparative longevity

Low levels of germination (<50%) were observed in seeds of *N. immutabilis*, *N. lukei*, *N. macrosperma* and *N. violacea* NV1 and NV4 for this experiment, precluding the fitting of seed survival curves to germination data for these species. However, for *N. violacea* NV2 and NV3, initial germination was *c.* 100%, with viability declining to 0% after 56 days of storage at 45°C and 60% RH. Fitting of seed survival curves to these data provided a *P_50_* of 19.5 (*Ki* = 2.27) and 24.8 (*Ki* = 2.62) days, for NV2 and NV3, respectively (Fig. 7).

### Moisture sorption isotherms

The relationship between seed water content and RH of seeds of all four species was found to be relatively sigmoidal in shape (Fig. 8). Seed moisture contents were similar for each species, over the range of RHs tested and ranged between *c.* 4–29% (*N. immutabilis*), 3–29% (*N. lukei*), 4–28% (*N. macrosperma*) and 3–28% *(N. violacea*).

### Seed lipid content

Seed lipid content was low in all species: *N. immutabilis* 2.8%, *N. lukei* NL2 3.5%, *N. macrosperma* NM2 3.1% and *N. violacea* NV2 1.1%, NV3 1.8% and NV4 1.9%.

### DSC

**Figure 9:**
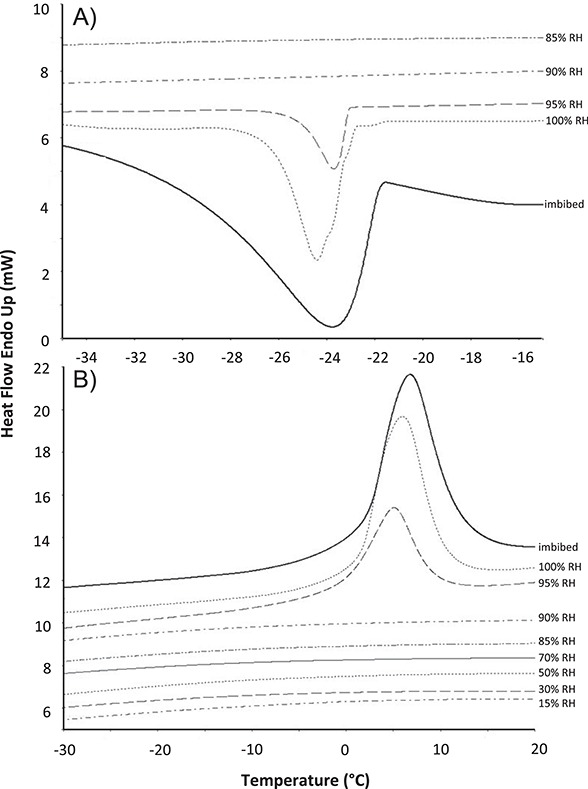
Representative (**A**) cooling and (**B**) warming thermograms of *N. violacea* (NV2) seeds equilibrated to 15–100% RH, or fully imbibed, prior to DSC analysis. Each sample was cooled to −50**°**C at 10**°**C/minute and rewarmed to 30**°**C 50**°**C/minute.

**Figure 10:**
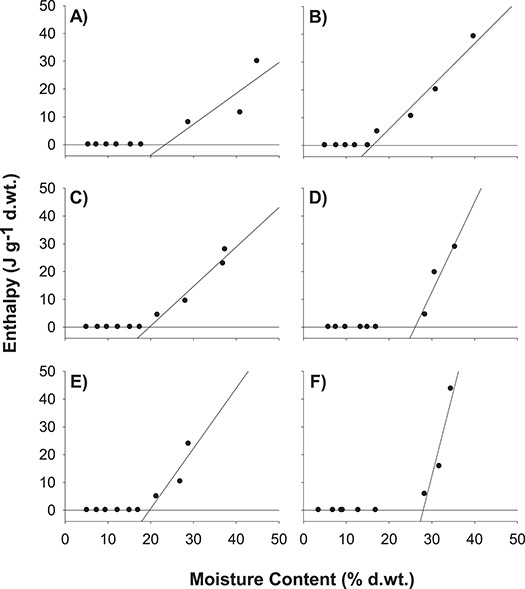
Determination of the UWC of (**A**) *N. immutabilis* (NI1; 23.4%), (**B**) *N. lukei* (NL2; 22.2%), (**C**) *N. macrosperma* (NM2; 19.7%) and (**D–F**) *N. violacea* (NV2 = 26.1%, NV3 = 19.9%, NV4 = 28.1%, respectively). Transition enthalpy was determined by calculating the area under the peak from warming thermograms.

DSC of seeds of *N. immutabilis*, *N. lukei*, *N. macrosperma* and *N. violacea* measured thermal transitions associated with water freezing and melting events (Fig. 9; Supplementary Table S4) for seeds at ≥90% RH. The onset temperature of the (exothermic) freezing peaks ranged between −14.2°C and −24.0°C, whilst melting (endothermic) onset temperature ranged between −2.9°C and 7.7°C. For seeds of all species equilibrated to ≤ 85% RH, no peaks were observed. Plotting of the enthalpy of the melting events against seed moisture content revealed the unfrozen moisture content varied somewhat between species (Fig. 10). Seeds of *N. macrosperma* had the highest unfrozen moisture content at 34.9%, whilst the unfrozen moisture content of *N. lukei* seeds was 16.2%, and for *N. immutabilis* was 23.4%. For *N. violacea* seeds the unfrozen moisture contents were determined to be 26.2% (NV2), 25.5% (NV3) and 28.0% (NV4). No phase changes associated with lipids or other non-water cellular components were evident during cooling or warming between −50°C and 30°C.

## Discussion

The objective of this study was to assess the desiccation sensitivity and *ex situ* seed storage behaviour of seeds from four species of tropical Australian *Nymphaea* to assist future conservation efforts associated with this iconic, basal, aquatic genus. Seeds from *N. immutabilis*, *N. lukei*, *N. macrosperma* and *N. violacea* were found to be tolerant of drying to 15% RH, or ~5% internal MC, and showed no significant decrease in viability immediately after drying. Desiccation tolerance appears to be a common trait amongst seeds of aquatic species (Dickie and Pritchard 2002), and our results, and those of Tuckett et al. (2010c) working with the Hydatellaceae, show the trait is present in basal, aquatic species inhabiting ephemeral wetland habitats. The species of *Nymphaea* we have examined here occur through the wet–dry tropics of northern Australia, which is a region characterized climatically by unpredictable cyclonic rainfall events over summer, followed by a period of drought during winter (Finlayson, 1999; Bureau of Meteorology, 2011). The ability of seeds to withstand drying presents a distinct advantage for aquatic species inhabiting ephemeral wetlands with transient water availability, allowing seeds to persist in the soil seedbank through the dry season (Dickie and Pritchard 2002). The contrasting desiccation sensitivity reported in seeds of *Nymphaea* species that occupy temperate and perennially inundated wetlands of the northern hemisphere (Guppy, 1897; Ewart, 1908; Smit *et al.*, 1989; Hay *et al.*, 2000; Estrelles *et al.*, 2004; Royal Botanic Gardens Kew, 2018) suggests desiccation sensitivity is a derived trait within these temperate Nymphaeaceae.

In terms of their comparative longevity, of the two collections of *N. violacea* we could fit viability curves to, the average *P_50_* was 22.2 days, which is broadly equivalent to that found for other Australian aquatics (Tuckett *et al.*, 2010b). However, in comparison with global datasets encompassing species from alpine regions, and dry, terrestrial environments where *P_50_* values range from 0.7 to 771 days (Probert *et al.*, 2009; Mondoni *et al.*, 2011; Merritt *et al.*, 2014; Satyanti *et al.*, 2018), it is apparent they sit within the shorter-lived end of the longevity spectrum. For the seed collections we were unable to fit viability curves to, the low levels of germination we observed may, at least in part, be attributable to seed dormancy, given the complex germination apparent in these species of *Nymphaea* (Dalziell *et al.*, 2018).

For the four species we have studied here, the responses of seeds to experimental storage conditions were complex and variable between collections of the same species. We consistently observed relatively low germination of seeds of *N. immutabilis*, *N. lukei* and *N. macrosperma*, even in freshly collected seeds, so we employed cut-testing and TZ staining to further inform on seed viability during storage, rather than rely on germination data alone. These viability data indicate seeds of all species were highly viable at the point of collection and prior to storage, but seeds of many species/accessions were short-lived across many of the storage treatments; albeit viability was maintained at ≥61% for 1 month, provided seeds were stored at low temperatures (−20°C or −190°C) and a pre-storage RH of 15%. For seeds stored at −190°C, viability in fact remained relatively stable across all RHs for at least 1 month. However, over a 12-month storage period, only seeds of three collections of *N. violacea* (NV2–NV4) remained highly viable (≥80%), and only when stored at 15% RH at temperatures < 0°C. For the most part, for seeds of NV2–NV4 viability declined with increasing pre-storage RH and increasing storage temperature in a manner consistent with seeds with orthodox storage behaviour. However, for seeds of the other collection of *N. violacea* (NV1), and seeds of *N. immutabilis*, *N. lukei*, and *N. macrosperma* (both collections), storage behaviour appeared more complex, with viability loss of seeds stored at −190°C equal to, or in many instances greater than, seeds stored at −20°C, or even at 5°C, after 6- or 12-months storage. Ultimately, between 40% and 100% viability was lost after 12-months storage for these collections. Thus, it is apparent for some or all collections of all species, decreasing the storage temperature had a limited to negligible effect on increasing longevity; seeds aged rapidly regardless of the storage conditions, and despite their desiccation tolerance.

Desiccation tolerant seeds that are short-lived and/or display complex storage behaviour represent a considerable challenge for conservation seed banking. Seeds that lose viability more quickly than might be expected at sub-zero temperatures are gaining more attention as data on storage of seeds of wild plant species continue to be accumulated (Walters 2015). Relatively poor longevity of cryostored seeds has been linked to seeds of low quality at the point of collection (Ballesteros and Pence 2017), but this does not appear to be a factor in our *Nymphaea* seeds. Our biophysical data also do not point to an obvious source of deterioration for seeds stored at sub-zero temperatures. DSC data determined the UWC was, whilst varied between species, such that ice crystal formation would have contributed to viability loss only for seeds stored at 95% RH, and ice formation is clearly not responsible for the significant loss of viability at RHs between 30–70% RH. Previous studies have linked the phase changes in triacylglycerol crystallization or melting during cooling and warming with the rapid deterioration during sub-zero temperature storage of oily seeds (and spores) in other species (Crane *et al.*, 2003, 2006; Ballesteros *et al.*, 2018). However, seeds of all *Nymphaea* species we have tested here possessed a very low oil content (1.1–3.7%), so damage induced by crystallization of storage lipids during cooling does not seem likely for *Nymphaea* seeds.

Seeds of the tropical members of *Nymphaea* studied here have complex germination behaviour; they possess either morphophysiological or physiological dormancy, and there is evidence of intraspecific variation in dormancy depth between seed populations (Dalziell *et al.*, 2018). It is apparent that the species that were more difficult to germinate (*N. immutabilis*, *N. lukei* and *N. macrosperma*) possessed more difficult storage behaviour. A similarly low initial germination response and ‘difficult’ germination behaviour has also been associated with short seed lifespan in some members of the Apiaceae (Walters, 2015). More complex germination behaviour has also been associated with low-temperature storage behaviour that departs from orthodox seeds in orchids (Hay et al. 2010), for which sensitivity to cryostorage was evident in a number of species, and seeds were better stored at −18°C. Any explicit relationships between complex dormancy/germination behaviour and seed storage behaviour are yet to be established, but is worthy of further exploration for seeds of diverse wild species.

With the ever-increasing anthropogenic pressures placed upon wild plants (Royal Botanic Gardens Kew, 2016), there is an increasing need to invest in *ex situ* conservation for wild species. Seeds of many Australian native species can be successfully stored *ex situ* under conventional conditions (Crawford *et al.*, 2007), but the seed storage behaviour of wild species in general is still relatively understudied (Walters, 2015), and for Australian species there is some increasing evidence of storage behaviour that departs from what might be expected of orthodox seeds (Hay *et al.*, 2010; Sommerville *et al.*, 2018). Our study on *Nymphaea* serves to highlight some of the inherent complexities and challenges associated with genebanking of wild seeds. It is clear that all four species will require some additional protocol development before we can have confidence that *ex situ* seed banking is a viable long-term germplasm conservation strategy. Whilst seeds of *N. violacea* may be successfully stored under conventional conditions for short periods of time, further investment should be made in refining the cryogenic storage protocols (Li and Pritchard 2009) to minimize viability decline and allow for the successful storage of seeds from *N. immutabilis*, *N. lukei* and *N. macrosperma*. As an alternative conservation strategy for these species of *Nymphaea*, conservation practitioners should also consider the feasibility of storing bulbs, rhizomes or tubers or the maintenance of whole plant living collections to ensure their survival *ex situ*.

## Supplementary Material

Dalziell_et_al_Nymphaea_seed_storage_supps_coz021
