# Supplementary material for: Seed storage behaviour of tropical members of the aquatic basal angiosperm genus Nymphaea L. (Nymphaeaceae)
Source: Conserv Physiol. 2019 May 10;7(1):coz021. doi: 10.1093/conphys/coz021 (PMC6510139; doi:10.1093/conphys/coz021)
Supplement: Dalziell_et_al_Nymphaea_seed_storage_supps_coz021 [file dalziell_et_al_nymphaea_seed_storage_supps_coz021.docx]

**SUPPLEMENTARY MATERIAL**

**Table S1:** Significance of regression terms of logistic regression (binomial) analysis and $6$time) following equilibration at 20**°**C and RH of 15, 30, 50, 70 or 90% at two storage temperatures (Te = -20**°**C and -190**°**C). Significance codes: P = *** < 0.001, ** < 0.01, * < 0.05.

|  |  | **Germination** | **Viability** |
| --- | --- | --- | --- |
| **Factor** | ***df*** | $\boldsymbol{\chi}^{\boldsymbol{2}}$ | $\boldsymbol{\chi}^{\boldsymbol{2}}$ |
| Seed collection | 8 | 5035.75*** | 1974.37*** |
| RH | 1 | 982.56*** | 712.78*** |
| Temperature | 4 | 118.15*** | 556.59*** |
| Time | 1 | 164.90*** | 1331.20*** |
| Seed collection x RH | 8 | 44.68*** | 86.78*** |
| Seed collection x Temperature | 17 | 335.93*** | 173.64*** |
| Seed collection x Time | 8 | 170.17*** | 684.11*** |
| RH x Temperature | 4 | 228.63*** | 227.43*** |
| RH x Time | 1 | 186.25*** | 370.23*** |
| Temperature x Time | 4 | 331.49*** | 345.95*** |

**Table S2:** Significance of regression terms of logistic regression (binomial) analysis and $\chi^{2}$ values for germination of nine collections of *Nymphaea* seeds stored for up to 12 months (Ti = time) following equilibration at 20**°**C and RH of 15, 30, 50, 70 or 90% at two storage temperatures (Te = -20**°**C and -190**°**C). Significance codes: P = *** < 0.001, ** < 0.01, * < 0.05.

| **Species and Collection** | **RH** | **Te** | **Ti** | **RH x Te** | **RH x Ti** | **Te x Ti** |
| --- | --- | --- | --- | --- | --- | --- |
| *N. immutabilis* (NI1) | 86.30*** | 5.64* | 0.42 | 1.36 | 26.47*** | 0.50 |
| *N. lukei* (NL1) | 123.61*** | 0.33 | 9.35** | 1.04 | 1.50 | 1.04 |
| *N. lukei* (NL2) | 29.51*** | 1.48 | 11.06*** | 3.10 | 4.84* | 2.26 |
| *N. macrosperma* (NM1) | 15.82*** | 0.74 | 63.72*** | 4.41* | 0.43 | 0.01 |
| *N. macrosperma* (NM2) | 59.60*** | 36.69*** | 59.98*** | 1.99 | 15.66*** | 57.03*** |
| *N. violacea* (NV1) | 161.81*** | 4.38* | 63.83*** | 1.66 | 1.52 | 0.03 |
| *N. violacea* (NV2) | 216.25*** | 1.07 | 42.15*** | 4.61* | 71.92*** | 18.04*** |
| *N. violacea* (NV3) | 177.32*** | 0.47 | 28.19*** | 10.13** | 41.65*** | 14.10*** |
| *N. violacea* (NV4) | 157.22*** | 167.20*** | 2.71 | 8.08** | 13.22*** | 11.24*** |

**Table S3:** Significance of regression terms of logistic regression (binomial) analysis and $\chi^{2}$ values for viability of nine collections of *Nymphaea* seeds stored for up to 12 months (Ti = time) following equilibration at 20**°**C and RH of 15, 30, 50, 70 or 90% at two storage temperatures (Te = -20**°**C and -190**°**C). Significance codes: P = *** < 0.001, ** < 0.01, * < 0.05.

| **Species and Collection** | **RH** | **Te** | **Ti** | **RH x Te** | **RH x Ti** | **Te x Ti** |
| --- | --- | --- | --- | --- | --- | --- |
| *N. immutabilis* (NI1) | 177.46*** | 11.87*** | 170.53*** | 11.41*** | 68.11*** | 5.48* |
| *N. lukei* (NL1) | 4.65* | <0.05 | 208.51*** | 3.36 | 7.42** | 1.80 |
| *N. lukei* (NL2) | 92.34*** | 0.25 | 414.30*** | 5.62* | 19.90*** | 21.97*** |
| *N. macrosperma* (NM1) | 0.91 | 10.95*** | 451.85*** | 0.08 | 0.27 | 0.27 |
| *N. macrosperma* (NM2) | 83.11*** | 11.15*** | 304.48*** | 0.83 | 50.29*** | 0.43 |
| *N. violacea* (NV1) | 28.18*** | 4.15* | 118.50*** | 0.08 | 0.75 | 29.09*** |
| *N. violacea* (NV2) | 141.10*** | 4.07* | 56.16*** | 0.38 | 116.06*** | 9.18** |
| *N. violacea* (NV3) | 115.86*** | 4.87* | 41.91*** | 0.10 | 72.26*** | 3.71 |
| *N. violacea* (NV4) | 157.51*** | 86.31*** | 101.01*** | 0.07 | 13.77*** | 17.68*** |

**Table S4:** Melting and warning peaks (°C) and total enthalpy (J/g) calculated from thermograms of whole seeds of *Nymphaea immutabilis, N. lukei, N. macrosperma* and *N. violacea*. Whole seeds were equilibrated to 15-100% RH or fully imbibed on irrigated glass filter paper prior to DSC analysis*.* Each sample was cooled to -50**°**C at 10**°**C/min and rewarmed to 30**°**C at a rate of 50**°**C/min. No enthalpy peaks were detected in any samples equilibrated to < 95% RH.

|  |  |  | **Cooling** | | | **Warming** | | | |
| --- | --- | --- | --- | --- | --- | --- | --- | --- | --- |
| **Species** | **Collection No.** | **Sample RH** | **Onset temp (°C)** | **Peak temp (°C)** | **Enthalpy (J/g)** | **Onset temp (°C)** | **Peak temp (°C)** | **Enthalpy (J/g)** |  |
| *N. immutabilis* | NI1 | 95 | -22.43 ± 1.32 | -23.16 ± 1.31 | -7.62 ± 0.68 | -0.77 ± 0.09 | 7.83 ± 0.45 | 9.77 ± 1.44 |  |
|  |  | 100 | -14.21 ± 0.86 | -14.82 ± 0.92 | -43.90 ± 4.35 | 3.49 ± 0.42 | 12.04 ± 1.21 | 43.45 ± 5.63 |  |
|  |  | Imbibed | -17.19 ± 0.65 | -18.39 ± 1.08 | -46.03 ± 4.35 | 7.74 ± 1.01 | 16.15 ± 0.83 | 13.65 ± 0.001 |  |
| *N. lukei* | NL1 | 95 | -22.99 ± 0.58 | -23.61 ± 0.64 | -19.08 ± 1.71 | -0.79 ± 0.56 | 7.31 ± 0.96 | 21.85 ± 1.59 |  |
|  |  | 100 | -12.93 ± 1.19 | -13.45 ± 1.17 | -32.73 ± 2.10 | 1.86 ± 0.28 | 7.40 ± 0.18 | 39.30 ± 1.88 |  |
|  |  | Imbibed | -19.67 ± 0.45 | - 22.81± 1.91 | -17.95 ± 2.43 | 3.69 ± 1.32 | 12.09 ± 2.34 | 15.27 ± 5.35 |  |
|  | NL2 | 95 | -24.25 ± 0.17 | -24.91 ± 0.17 | -7.62 ± 1.14 | 0.48 ± 0.99 | 6.26 ± 0.29 | 10.19 ± 1.66 |  |
|  |  | 100 | -17.45 ± 0.46 | -17.85 ± 0.47 | -58.76 ± 11.03 | 1.65 ± 0.36 | 7.05 ± 0.05 | 56.04 ± 10.51 |  |
|  |  | Imbibed | -20.81 ± 1.99 | -21.90 ± 2.49 | -37.31 ± 11.52 | 3.89 ± 0.12 | 11.23 ± 1.33 | 34.18 ± 10.35 |  |
| *N. macrosperma* | NM1 | 95 | -24.04 ± 0.26 | -24.93 ± 0.21 | -4.50 ± 1.88 | -1.87 ± 1.36 | 7.65 ± 2.23 | 6.09 ± 1.36 |  |
|  |  | 100 | -16.08 ± 0.22 | -15.29 ± 1.24 | -33.39 ± 0.85 | 3.17 ± 0.62 | 12.12 ± 1.15 | 35.98 ± 3.09 |  |
|  |  | Imbibed | -14.48 ±1.42 | -15.54 ± 1.53 | -41.47 ± 6.99 | 6.28 ± 0.75 | 14.16 ± 0.68 | 24.97 ± 6.63 |  |
|  | NM2 | 95 | -17.42 ± 1.36 | -18.03 ± 1.36 | -28.76 ± 10.83 | -1.02 ± 0.83 | 6.06 ± 0.82 | 32.49 ± 12.99 |  |
|  |  | 100 | -16.88 ± 1.07 | -17.34 ± 1.06 | -59.68 ± 12.70 | 2.33 ± 0.26 | 9.03 ± 0.84 | 55.76 ± 10.97 |  |
|  |  | Imbibed | -18.45 ± 1.45 | -21.16 ± 0.96 | -51.46 ± 4.54 | 3.49 ± 0.72 | 11.64 ± 0.65 | 43.78 ± 4.46 |  |
| *N. violacea* | NV1 | 95 | -21.37 ± 1.44 | -22.00 ± 1.52 | -15.84 ± 6.10 | -1.00 ± 1.13 | 5.89 ± 1.57 | 19.03 ± 6.29 |  |
|  |  | 100 | -21.49 ± 0.32 | -24.12 ± 0.26 | -30.67 ± 6.09 | 0.91 ± 0.34 | 5.89 ± 0.06 | 38.19 ± 16.71 |  |
|  |  | Imbibed | -22.75 ± 0.35 | -23.38 ± 0.23 | -27.67 ± 5.22 | 1.01 ± 0.63 | 6.69 ± 0.10 | 28.97 ± 4.74 |  |
|  | NV2 | 95 | -21.59 ± 1.71 | -22.31 ± 1.87 | -17.95 ± 5.01 | -2.93 ± 0.13 | 4.47 ± 0.58 | 25.60 ± 8.34 |  |
|  |  | 100 | -16.67 ± 0.86 | -20.64 ± 1.14 | -15.54 ± 4.68 | 0.98 ± 1.44 | 6.54 ± 1.11 | 18.37 ± 3.41 |  |
|  |  | Imbibed | -22.87 ± 0.23 | -24.16 ± 0.25 | -12.05 ± 0.86 | 1.21 ± 0.14 | 9.71 ± 0.61 | 14.38 ± 0.31 |  |
|  | NV3 | 95 | -23.22 ± 0.45 | -23.73 ± 0.76 | -12.03 ± 1.15 | -0.10 ± 0.45 | 5.20 ± 0.31 | 10.17 ± 2.31 |  |
|  |  | 100 | -18.56 ± 1.12 | -18.99 ± 0.97 | -38.66 ± 5.22 | 0.60 ± 0.11 | 6.48 ± 0.48 | 51.04 ± 9.46 |  |
|  |  | Imbibed | -23.24 ± 0.12 | -24.30 ± 0.33 | -24.41 ± 0.05 | 2.03 ± 0.90 | 9.59 ± 2.29 | 24.58 ± 1.26 |  |
|  | NV4 | 95 | -22.51 ± 0.91 | -23.12 ± 0.90 | -5.77 ± 1.21 | -0.37 ± 0.25 | 5.22 ± 0.25 | 7.02 ± 1.54 |  |
|  |  | 100 | -18.36 ± 0.78 | -18.79 ± 0.34 | -50.63 ± 5.13 | 1.95 ± 0.56 | 6.30 ± 0.34 | 61.19 ± 4.39 |  |
|  |  | Imbibed | -17.38 ± 1.43 | -18.25 ± 1.43 | -47.38 ± 15.61 | 3.06 ± 0.65 | 10.26 ± 1.03 | 44.25 ± 14.04 |  |
